# Transcription factor-based biosensors in biotechnology: current state and future prospects

**DOI:** 10.1007/s00253-015-7090-3

**Published:** 2015-10-31

**Authors:** Regina Mahr, Julia Frunzke

**Affiliations:** Institute of Bio- and Geosciences, IBG-1: Biotechnology, Forschungszentrum Jülich GmbH, 52425 Jülich, Germany

**Keywords:** Transcriptional regulator, Biosensor, Metabolic engineering, Screening, Evolution, Single-cell analysis

## Abstract

Living organisms have evolved a plethora of sensing systems for the intra- and extracellular detection of small molecules, ions or physical parameters. Several recent studies have demonstrated that these principles can be exploited to devise synthetic regulatory circuits for metabolic engineering strategies. In this context, transcription factors (TFs) controlling microbial physiology at the level of transcription play a major role in biosensor design, since they can be implemented in synthetic circuits controlling gene expression in dependency of, for example, small molecule production. Here, we review recent progress on the utilization of TF-based biosensors in microbial biotechnology highlighting different areas of application. Recent advances in metabolic engineering reveal TF-based sensors to be versatile tools for strain and enzyme development using high-throughput (HT) screening strategies and adaptive laboratory evolution, the optimization of heterologous pathways via the implementation of dynamic control circuits and for the monitoring of single-cell productivity in live cell imaging studies. These examples underline the immense potential of TF-based biosensor circuits but also identify limitations and room for further optimization.

## Introduction

In the last century, the era of metabolic engineering resulted in an enormous increase in microbial processes for the production of value-added compounds, such as proteins, amino acids, biofuels, organic acids and polymer precursors. Based on renewable feedstocks, the efficient establishment and optimization of bioprocesses is the key to a transition from the currently petroleum-dependent and energy-intensive chemical industry towards a sustainable bioeconomy.

**Fig. 1:**
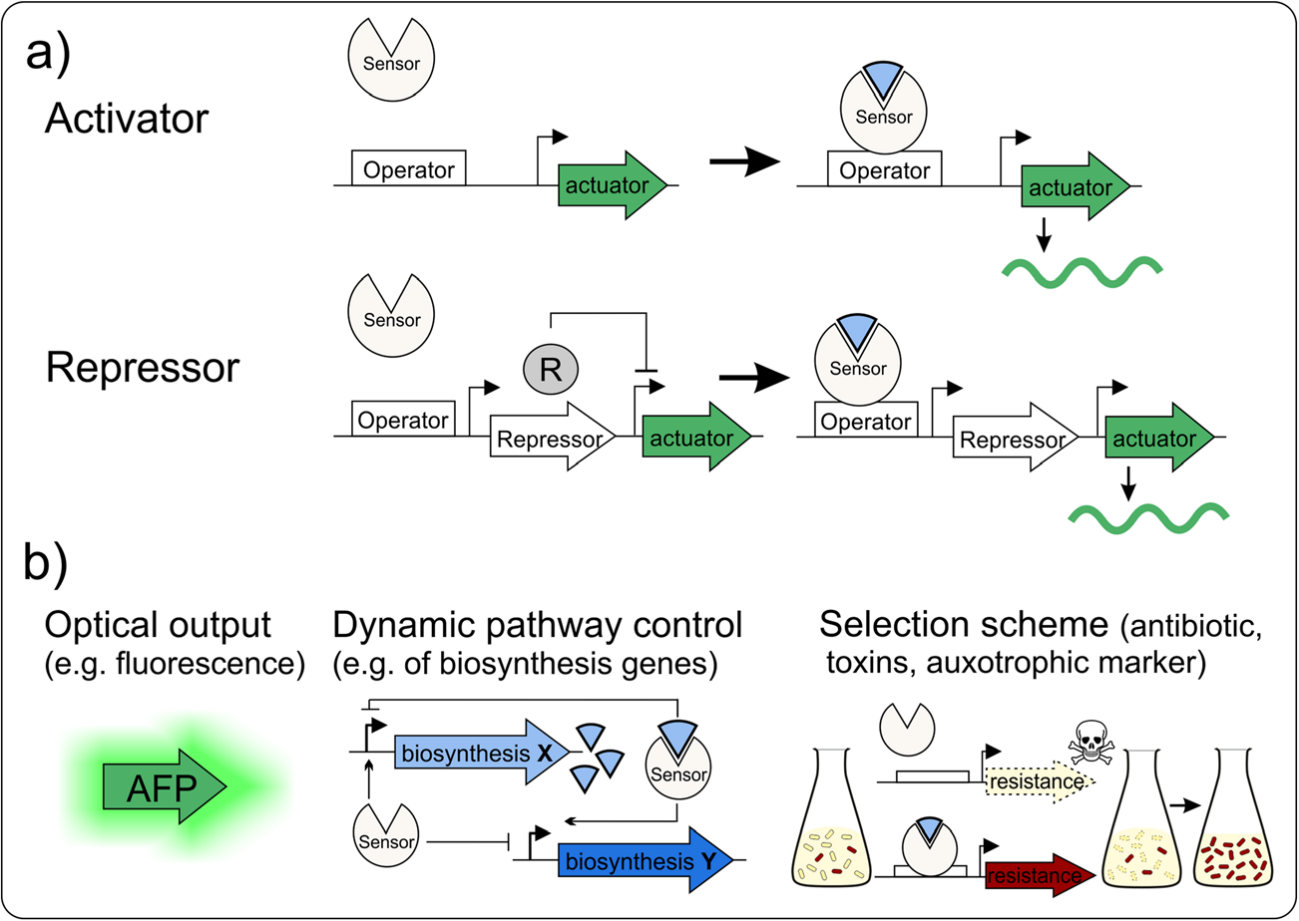
Principles for the architecture of transcription factor-based biosensors. **a** A transcriptional activator may be used to activate expression of an actuator gene (circuit) in response to effector molecules. In contrast, repressors block the expression of actuators. By setting the expression of a second repressor under the control of the TF-biosensor repressor, the signalling can be inverted, resulting in a positive output of the actuator module. **b** Depending on the final function, different actuators are available as biosensor readout. The expression of e.g. autofluorescent proteins (AFP) results in an optical output, while the insertion of the biosensor into regulatory circuits can trigger and dynamically control biosynthetic pathways. Sensors can further be used to generate an artificial selection scheme by the choice of a suitable actuator (e.g. antibiotics, toxins or auxotrophy) controlling the survival of strains with desired traits

Exploiting microorganisms for large-scale production requires, on the one hand, elaborated high-throughput (HT) tools for strain engineering, and, on the other hand, techniques for analyzing the performance of producer strains and the efficiency of bioprocesses. Recent studies using metabolic flux analysis and in silico modelling approaches enable new insights into the bacterial physiology during fermentation (Wiechert and Noack 2011); however, the formation of inefficient subpopulations affecting the outcome of the bioprocess is often neglected (Delvigne and Goffin 2014; Lieder et al. 2014). While rational strain engineering is limited by the high physiological complexity of microbes, traditional random mutagenesis strategies are restricted by the selection and screening capacity, which requires a readily accessible phenotype linked to product formation (Dietrich et al. 2010; Schallmey et al. 2014). During the past decade, advances in synthetic biology significantly contributed to the establishment of novel metabolic engineering tools (Ng et al. 2015; Wendisch 2014). For example, genetically encoded biosensors have proven to be of high value for various applications in strain engineering, dynamic pathway control and single-cell analysis. The basic principle is based on metabolite-sensing proteins (e.g. transcription factors, enzymes or periplasmic-binding proteins) or RNAs (e.g. riboswitches and ribozymes) which are activated upon binding of effector molecules and control in turn the expression of an actuator part (e.g. fluorescent reporters, regulatory switches or selection markers). This biosensor architecture enables the intracellular detection of metabolite production by converting it into a measureable output (Fig. 1).

**Fig. 2:**
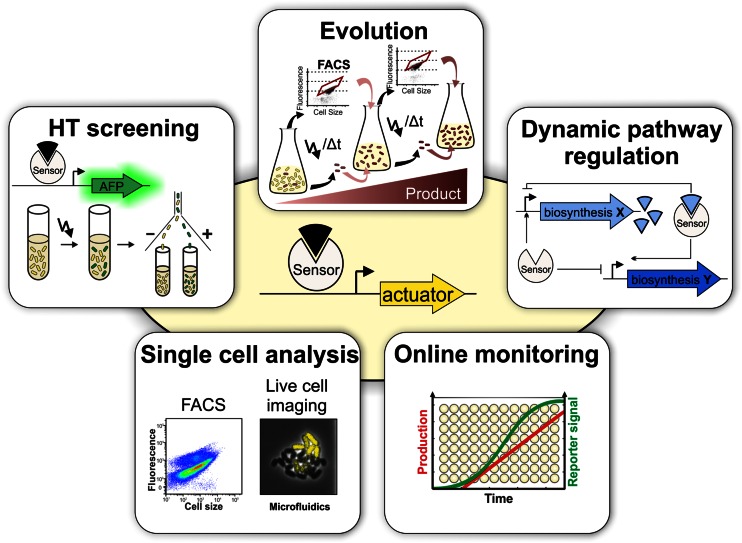
Versatile applications of TF-based biosensors. Biosensors with an optical readout, e.g. production of an autofluorescent protein (*AFP*), are efficient tools for the high-throughput (*HT*) screening of large mutant libraries using fluorescence-activated cell sorting (*FACS*). Biosensor-driven evolution has proven a convenient strategy to increase production by iteratively imposing an artificial selective pressure on the fluorescent output of a biosensor using FACS or selection schemes. Integrated into synthetic regulatory circuits, biosensors can be used for the dynamic control of biosynthetic pathways in order to avoid, for example, the accumulation of toxic intermediates. Finally, biosensors are convenient tools for non-invasive online monitoring of production processes and for analysis at single-cell resolution using FACS and live cell imaging in microfluidic chip devices

In the following sections, we will review recent progress regarding the design of biosensor circuits based on transcription factors (TFs) and their application in metabolic engineering strategies including HT screening approaches, dynamic pathway control, biosensor-driven evolution and single-cell analysis (Fig. 2). We will not include the application of TF-based biosensors for the detection of environmental pollutants, which is reviewed elsewhere (Fernandez-Lopez et al. 2015; van der Meer and Belkin 2010). For recent review articles on RNA- and FRET-based biosensors, see Frommer et al. (2009), Liang et al. (2011), Michener et al. (2012), Schallmey et al. (2014) and Zhang et al. (2015).

## Exploiting nature’s toolbox—transcription factor-based biosensors

Living organisms have evolved a variety of different sensor principles to monitor the intra- or extracellular accumulation of small molecules, ions or changes in physical parameters. In prokaryotes, TFs play a major role in physiological adaptation by controlling gene expression at the level of transcription—typically by interfering with the binding of the RNA polymerase to DNA. The activity of TFs can be affected by the interaction with small (effector) molecules, ions, physical parameters (e.g. temperature or pH), protein-protein interaction or protein modification. In several recent studies, researchers have demonstrated that these mechanisms provide a versatile toolbox for applications in metabolic engineering and single-cell analysis of production strains (Table 1) (Liu et al. 2015a; Michener et al. 2012; Schallmey et al. 2014).

**Table 1:** Overview of TF-based biosensors applied in biotechnological strain development and screening approaches

TF	Analyte	Host chassis	Output	Application
AraC-Idi_Synth_; based on AraC of *E. coli*	Isopentenyl diphosphate (lycopene)	*E. coli*	MutD5-mCherry	Improvement of isopentenyl diphosphate production of *E. coli* using a biosensor-controlled mutator strategy. Visualization of the production by the biosensor output (Chou and Keasling 2013)
BenR of *P. putida*	Benzoate	*E. coli*	GFP	Screening of a metagenomics library for improved amidase activities (Uchiyama and Miyazaki 2010a)
BmoR of *Thauera butanivorans*	1-Butanol (response to linear and branched-chain alcohols)	*E. coli*	TetA-GFP	Improvement of 1-butanol production of *E. coli* by a biosensor-based selection scheme. Simultaneous monitoring of growth and fluorescence as measure of the biosensor output (Dietrich et al. 2013)
CysR of *C. glutamicum*	O-acetyl (homo-) serine	*C. glutamicum*	eYFP	Visualization of sulphur limitation at the single cell level (Hoffmann et al. 2013)
DcuR of *E. coli*	Succinate	*E. coli*	TetA	Proof-of-concept study: linking dicarboxylic acid production to bacterial growth (Dietrich et al. 2013)
FadR of *E. coli*	Fatty acid/acyl-CoA	*E. coli*	RFP/regulatory circuit	Implementation of a synthetic circuit for dynamic pathway control of the production of fatty acid ethyl ester in *E. coli* (Zhang et al. 2012)
FapR of *B. subtilis*	Malonyl-CoA	*E. coli*	eGFP/regulatory circuit	• Design and kinetic analysis of a malonyl-CoA sensor in *E. coli* (Xu et al. 2014b) • TF-based negative feedback loop for the dynamic control of fatty acid biosynthesis in dependency of the intracellular malonyl-CoA level (Liu et al. 2015b)
LacI of *E. coli*	IPTG, lactose	*E. coli*	GFP	Live cell imaging study of the correlation between growth rate fluctuations and metabolic stochasticity (Kiviet et al. 2014)
Lrp of *C. glutamicum*	L-valine L-leucine L-isoleucine L-methionine	*C. glutamicum*	eYFP	• HT FACS screening of a chemically mutagenized *C. glutamicum* wt library (Mustafi et al. 2012) • Live cell imaging of L-valine production of PDHC-deficient *C. glutamicum* strains (Mustafi et al. 2014) • Biosensor-driven evolution of L-valine production (Mahr et al. 2015)
LysG of *C. glutamicum*	L-lysine L-arginine L-histidine	*C. glutamicum*	eYFP	• HT FACS screening of a chemically mutagenized *C. glutamicum* wt library (Binder et al. 2012) • Screening of enzyme libraries for feedback-resistant variants of key enzymes for amino acid production (Schendzielorz et al. 2014)
NahR of *P. putida*	Benzoic acids	*E. coli*	TetA	Proof-of-concept study: selection of biocatalysts by the implementation of a TF-based selection scheme (van Sint Fiet et al. 2006)
PcaR of *P. putida*	ß-ketoadipate	*E. coli*	TetA	Proof-of-concept study: linking ß-ketoadipate production to bacterial growth (Dietrich et al. 2013)
SoxR of *E. coli*	NADPH	*E. coli*	eYFP	HT FACS screening of a mutant library of the NADPH-dependent alcohol dehydrogenase of *Lactobacillus brevis* for improved 4-methyl-2-pentanone (Siedler et al. 2014a)
TyrR of *E. coli*	L-tyrosine	*E. coli*	MutD5-mCherry	Improvement of L-tyrosine production of *E. coli* using a biosensor-controlled mutator strategy. Visualization of the production by the biosensor output (Chou and Keasling 2013)

Especially, metabolite-responsive TFs have proven to be valuable tools for biotechnological applications and have been integrated into a diverse set of synthetic regulatory circuits enabling the detection of, for example, amino acids (Binder et al. 2012; Mustafi et al. 2012), succinate (Dietrich et al. 2013), butanol (Dietrich et al. 2013), malonyl-CoA (Xu et al. 2014a, b) and secondary metabolites (Siedler et al. 2014b). These circuits are typically based on a previously well-characterized TF which limits the rapid access to novel metabolite sensors to a small set of known TFs. However, the principle of substrate-induced gene expression (SIGEX), where fragments of a metagenomic library can be ligated into an operon-trap vector in front of a suitable reporter gene (e.g. *gfp*), might represent an option to overcome this limitation (Uchiyama and Miyazaki 2010b; Uchiyama and Watanabe 2008). Originally developed for the screening of novel enzymes and biosynthetic operons, this design can in principle also be exploited to screen metagenomic libraries for effector-responsive TF-promoter pairs. Furthermore, global databases like DBD (www.transcriptionfactor.org; (Wilson et al. 2008)), RegPrecise (http://regprecise.lbl.gov/RegPrecise; (Novichkov et al. 2013)) or PRODORIC (www.prodoric.de; (Münch et al. 2003)) are useful tools to gain information on prokaryotic transcription factors and regulons. Finally, plenty of species-specific databases are available, including RegulonDB (http://regulondb.ccg.unam.mx; (Salgado et al. 2006)) and EcoCyc (http://ecocyc.org; (Keseler et al. 2013)) for *Escherichia coli* or CMRegNet (www.lgcm.icb.ufmg.br/cmregnet; (Abreu et al. 2015)) for corynebacterial and mycobacterial species which also provide valuable information regarding regulatory circuits for the development of novel sensor devices.

Besides classical one-component TFs, the principle of two-component signalling (TCS) represents a promising mode for the extracellular detection of small molecules in production strains or synthetic communities. Previous studies have already demonstrated that the modular design of TCS can be exploited to create sensor kinases with novel effector specificities and to transduce the information to the level of gene expression (Ohlendorf et al. 2012). In a recent study, Ganesh and co-workers reported on the construction of a chimeric, malate-responsive TCS by fusing the sensor domain of MalK (*Bacillus subtilis*) to the kinase domain of EnvZ (*Escherichia coli*) thereby controlling the activity of the *ompC* promoter in response to external malate accumulation (Ganesh et al. 2015). To ensure specific signal transduction and to avoid detrimental cross-talk to host TCSs, the stoichiometry, the expression level of the protein components, as well as the potential phosphatase activity of the sensor kinase remain critical aspects to be considered for the design of TCS-based biosensors (Podgornaia and Laub 2013).

An alternative principle for intra- or extracellular sensing is represented by extracytoplasmic function (ECF) sigma factors (Mascher 2013). The orthogonality of ECF-based switches has recently been demonstrated by a proof-of-principle study describing the construction of a bistable switch in *E. coli* (Chen and Arkin 2012) and was further developed by Rhodius et al., who characterized ECF sigma factor families in bacteria using bioinformatics. The authors reported on 20 highly orthogonal combinations of sigma factors and their cognate promoters (Rhodius et al. 2013). These studies provide a promising basis for the design of synthetic circuits in metabolic engineering.

## High-throughput screening

Genetically encoded biosensors enable the specific translation of intracellular product accumulation into a screenable (e.g. fluorescence) or selectable (e.g. antibiotic resistance) output by driving the production of a reporter protein (Fig. 1b). Consequently, an important field of biosensor application is implementation in HT screening approaches for the selection of novel or improved biocatalysts (Fig. 2) (Eggeling et al. 2015; Schallmey et al. 2014). Fluorescence-activated cell sorting (FACS) was applied in several recent studies as a particularly suitable HT technique. For example, the transcriptional regulator Lrp of *Corynebacterium glutamicum* was recently implemented in a FACS HT screening approach for the isolation of mutant strains producing branched-chain amino acids (L-valine, L-leucine and L-isoleucine) from a mutant library after chemical mutagenesis (Mustafi et al. 2012). The native function of Lrp is to sense the intracellular accumulation of branched-chain amino acids and methionine, and in turn to activate the amino acid export system BrnFE in order to avoid high intracellular levels and toxic effects of these amino acids (Lange et al. 2012). These characteristics provide an optimal basis for the construction of biosensors featuring an appropriate dynamic range and sensitivity for the improvement of production strains. In addition, they have a significant advantage in comparison to the use of sensors based on transcriptional (biosynthesis) repressors or periplasmic-binding proteins, which typically display a very high effector affinity. The successful application of a similar activator protein has also been demonstrated by a study using the LysG TF for the isolation of L-lysine-producing strains of *C. glutamicum* via FACS (Binder et al. 2012).

Furthermore, TF-based sensors were successfully exploited in enzyme screenings. For example, the abovementioned LysG sensor was used to screen enzyme libraries for feedback-resistant enzyme variants for the overproduction of the effector amino acids L-arginine (N-acetyl-L-glutamate kinase), L-histidine (ATP phosphoribosyl transferase) and L-lysine (aspartate kinase) (Schendzielorz et al. 2014). An engineered AraC variant was used by Tang and co-workers for the directed evolution of 2-pyrone synthase activity (from *Gerbera hybrida*) in *E. coli*. Two iterative rounds of mutagenesis and selection led to the isolation of enzyme variants displaying roughly 20-fold increased triacetic acid lactone production (Tang et al. 2013). The considerable plasticity of the AraC protein for the engineering of new effector specificities was already previously demonstrated in a study where a mevalonate-responsive AraC variant was used for the screening of ribosome binding site (RBS) variants in front of a hydroxymethylglutaryl-CoA reductase (Tang and Cirino 2011). A promising alternative to the sensing of product formation was recently demonstrated by the application of an NADPH-responsive biosensor based on *E. coli* SoxR. This sensor provides a broadly applicable tool for the screening of NADPH-dependent enzymes, as exemplified by screening a dehydrogenase library for enzymes exhibiting improved catalytic activity for the substrate 4-methyl-2-pentanone (Siedler et al. 2014a).

As an alternative to screening strategies, TF-based biosensors can also be integrated in circuits to establish a product-dependent selection scheme driving the expression of, for example, an antibiotic resistance or toxin gene (Fig. 1b) (Dietrich et al. 2013; Raman et al. 2014; van Sint Fiet et al. 2006). The proof-of-principle was provided by a study of van Sint Fiet et al., who used the transcriptional activator NahR which responds to benzoate and 2-hydroxybenzaldehyde by the activation of *tetA* (or *lacZ*) expression (van Sint Fiet et al. 2006). The authors suggested that this design enables the efficient selection of novel or improved biocatalysts for chemical synthesis. Suitability of such a circuit design was later, for instance, demonstrated by the improvement of 1-butanol production of engineered *E. coli* by using the putative σ^54^-transcriptional activator BmoR and a σ^54^-dependent, alcohol-regulated promoter (P_BMO_) from *Pseudomonas butanovora* driving the expression of a *tetA*-*gfp* gene fusion (Dietrich et al. 2013). This setup allowed the simultaneous monitoring of growth and fluorescence as a measure of the biosensor output.

## Dynamic pathway control

In microorganisms, small molecule biosynthesis is typically controlled by a complex regulatory network which optimizes metabolic flux according to the requirements of the host and counteracts the accumulation of toxic intermediates. Consequently, the simple integration of heterologous biosynthetic pathways or enzymes may lead to unbalanced flux and detrimental interference with the host metabolism. In this context, TF-based biosensors can be used to construct synthetic regulatory switches to dynamically regulate metabolic fluxes (Figs. 1b and 2). This has, for example, been achieved by using the fatty acyl-CoA biosensor FadR to coordinate the biosynthesis of acyl-CoA and ethanol as well as the expression of a wax-ester synthase in an *E. coli* strain producing fatty acid ethyl ester (FAEE) (Zhang et al. 2012). Upon accumulation of acyl-CoA, the repressor FadR dissociates from its target promoters, leading to the activation of ethanol biosynthesis and the expression of wax-ester synthase, which converts ethanol and acyl-CoA to FAEE. Similarly, Xu and co-workers designed a hybrid promoter-regulator system based on the malonyl-CoA-responsive TF FapR in *E. coli* (Xu et al. 2014b). This regulator was further used to devise different negative feedback loops for the dynamic control of the enzymes acetyl-CoA carboxylase and fatty acid synthase for improved fatty acid biosynthesis as a function of intracellular malonyl-CoA levels (Liu et al. 2015b; Xu et al. 2014a).

The fact that accumulation of toxic intermediates may lead to a complex cellular stress response can also be exploited for the design of synthetic circuits balancing the pathway flux. In contrast to the choice of a well-known TF for circuit design, transcriptome analysis by DNA microarrays or RNA-Seq may be applied to uncover genes whose expression is altered upon accumulation of a certain pathway intermediate. For instance, exploiting the cellular response of *E. coli* to the accumulation of farnesyl pyrophosphate was used to balance terpenoid production (Dahl et al. 2013). However, transcriptome analysis provides a snapshot view of the cellular response to metabolite accumulation and, thus, the dynamic behaviour of the particular transcriptional response can hardly be estimated. Furthermore, complex regulatory hierarchies will likely hinder the exact description of the sensor transfer curve and its application for the dynamic control of heterologous pathways.

## Biosensor-driven adaptive evolution

Due to the high physiological complexity of living organisms and the limited knowledge of their underlying mechanisms, alternative approaches are in demand to efficiently engineer bacterial strains for biotechnological applications. Random mutagenesis strategies, however, lead to several hundred undirected small nucleotide polymorphisms (SNPs) genome-wide (Harper and Lee 2012), which makes it difficult to identify mutations contributing to the desired phenotypic trait. Evolution approaches driven by mutation and selection have proven a valuable tool to adapt microorganisms to stress conditions (Lee et al. 2013; Oide et al. 2015) or to improve product formation (Reyes et al. 2014; Xie et al. 2015). In several recent strategies, biosensors were successfully implemented to expand adaptive laboratory evolution to include production phenotypes which are not naturally linked to bacterial growth or fitness (Fig. 2) (Chou and Keasling 2013; Dietrich et al. 2013; Mahr et al. 2015; Yang et al. 2013).

Using feedback-regulated evolution of phenotype (FREP), Chou and Keasling dynamically regulated the mutation rate of a strain defective in the DNA repair machinery by controlling the mutator gene (*mutD5*) as the actuator of a small molecule biosensor (Chou and Keasling 2013). The FREP strategy was successfully applied in *E. coli* to increase tyrosine production up to fivefold. Using the same strategy, the propagation of high lycopene producer cells for a total cultivation of 432 h yielded up to 6800 μg lycopene g^−1^ dry cell weight. The application of FREP, however, resulted in several hundred SNPs throughout the entire genome (Chou and Keasling 2013). To reduce the number of mutations, we recently established a biosensor-driven adaptive evolution strategy, which is based on the natural mutation frequency of 10^−10^ to 10^−9^ mutations per base pair per replication cycle (Mahr et al. 2015). Using FACS, cells exhibiting a high biosensor output (eYFP fluorescence) were iteratively isolated and recultivated. Within five rounds of evolution, growth and the L-valine product formation of a pyruvate-dehydrogenase-deficient *C. glutamicum* strain were significantly improved, while at the same time a three- to fourfold reduction in by-product (L-alanine) formation was achieved. Four out of seven identified SNPs were reintroduced into the parental strain and were found to significantly increase L-valine production or to reduce by-product formation (Mahr et al. 2015).

Since artificial selection schemes may result in the enrichment of (false positive) cheaters, Raman et al. devised a combination of a positive and negative selection strategy based on the TolC selector (positive selection: sodium dodecyl-sulphate; negative selection: using colicin E1, (DeVito 2008)). This elegant design enabled the performance of multiple toggled rounds of selection to improve the production of naringenin and glucaric acid (Raman et al. 2014). Altogether, these examples demonstrate that biosensor-driven evolution represents a suitable strategy to complement rational approaches for the engineering of production strains.

## Single-cell analysis

Microbial metabolism is typically analyzed using bulk techniques neglecting single-cell behaviour and the formation of complex phenotypic patterns (Huang 2009; Vasdekis and Stephanopoulos 2015). However, even clonal groups of microorganisms may display significant phenotypic variation which can significantly contribute to the fitness of the whole population in its natural ecological niche (Ackermann 2015). Cell-to-cell variability caused by intrinsic or extrinsic factors may, however, strongly influence bioprocess performance and stability (Delvigne et al. 2014; Müller et al. 2010). The formation of inefficient subpopulations has, for example, been observed in the production of solvent by endospore-forming *Clostridia* (Tracy et al. 2008), the production of lactobionic acid in *Pseudomonas taetrolens* (Alonso et al. 2012) and the production of heterologous proteins by *E. coli* (Want et al. 2009), *Bacillus megaterium* (Münch et al. 2015) and yeast (Carlquist et al. 2012; Newman et al. 2006). However, only a limited number of studies implemented TF-based biosensors for single-cell analysis of production strains, so far (Delvigne et al. 2009; Hoffmann et al. 2013; Mustafi et al. 2014).

Recent advances in live cell imaging approaches using microfluidic chip devices and flow cytometry (FC) have significantly contributed to the analysis and monitoring of microbial populations at single-cell resolution (Fig. 2) (Delvigne and Goffin 2014; Grünberger et al. 2014; Vasdekis and Stephanopoulos 2015). To address the variety of biological questions, different microfluidic chips have recently been developed for the spatiotemporal analysis of microbial populations, including two-dimensional picolitre bioreactor chambers (Grünberger et al. 2012, 2014) as well as one-dimensional designs (e.g. the mother machine (Long et al. 2013; Wang et al. 2010)) for the long-term study of bacterial growth and fluorescence. The mother machine structure was, for instance, applied to analyze the correlation of growth rate fluctuations and metabolic stochasticity using a LacI-sensor (Kiviet et al. 2014). In this study, Kiviet and co-workers demonstrated how gene expression noise can affect growth rate fluctuations and vice versa, leading to cellular heterogeneity (Kiviet et al. 2014). Recently, the abovementioned Lrp biosensor was applied to monitor L-valine production of pyruvate-dehydrogenase-deficient *C. glutamicum* strains grown in 2D microfluidic chip devices (Mustafi et al. 2014). Interestingly, the addition of small amounts of complex medium compounds, as often used during production processes, resulted in phenotypic heterogeneity during the production phase (Mustafi et al. 2014).

Complementing live cell imaging studies, FC allows the convenient analysis of populations grown in large volumes such as shake flasks or bioreactors by HT processes (Huang 2009; Vasdekis and Stephanopoulos 2015). Combined with biosensors, FC has the potential to identify the formation of subpopulations with respect to metabolic activity, co-factor supply or cell cycle state and to use this information for the optimization of bioprocesses. For example, Delvigne and co-workers revealed subpopulations differing in *rpoS* expression applying oscillating feed control during fermentation using a transcriptional *rpoS-gfpmut2* sensor construct (Delvigne et al. 2009). Furthermore, recent advances in the establishment of downstream analytical methods bring the analysis of isolated subpopulations within reach. Jehmlich and co-workers established a workflow to analyze the proteome of FACS-isolated subpopulations by mass spectrometry (Jahn et al. 2013; Jehmlich et al. 2010). This protocol was successfully applied to analyze subpopulations occurring during the growth of *Pseudomonas putida* KT2440 in bioprocesses (Lieder et al. 2014). Altogether, these examples highlight the recent advances in single-cell analysis of microbial production strains. Combined with TF-based biosensors, these technological advances will significantly increase the resolution of bioprocess monitoring.

## Biosensor engineering

**Fig. 3:**
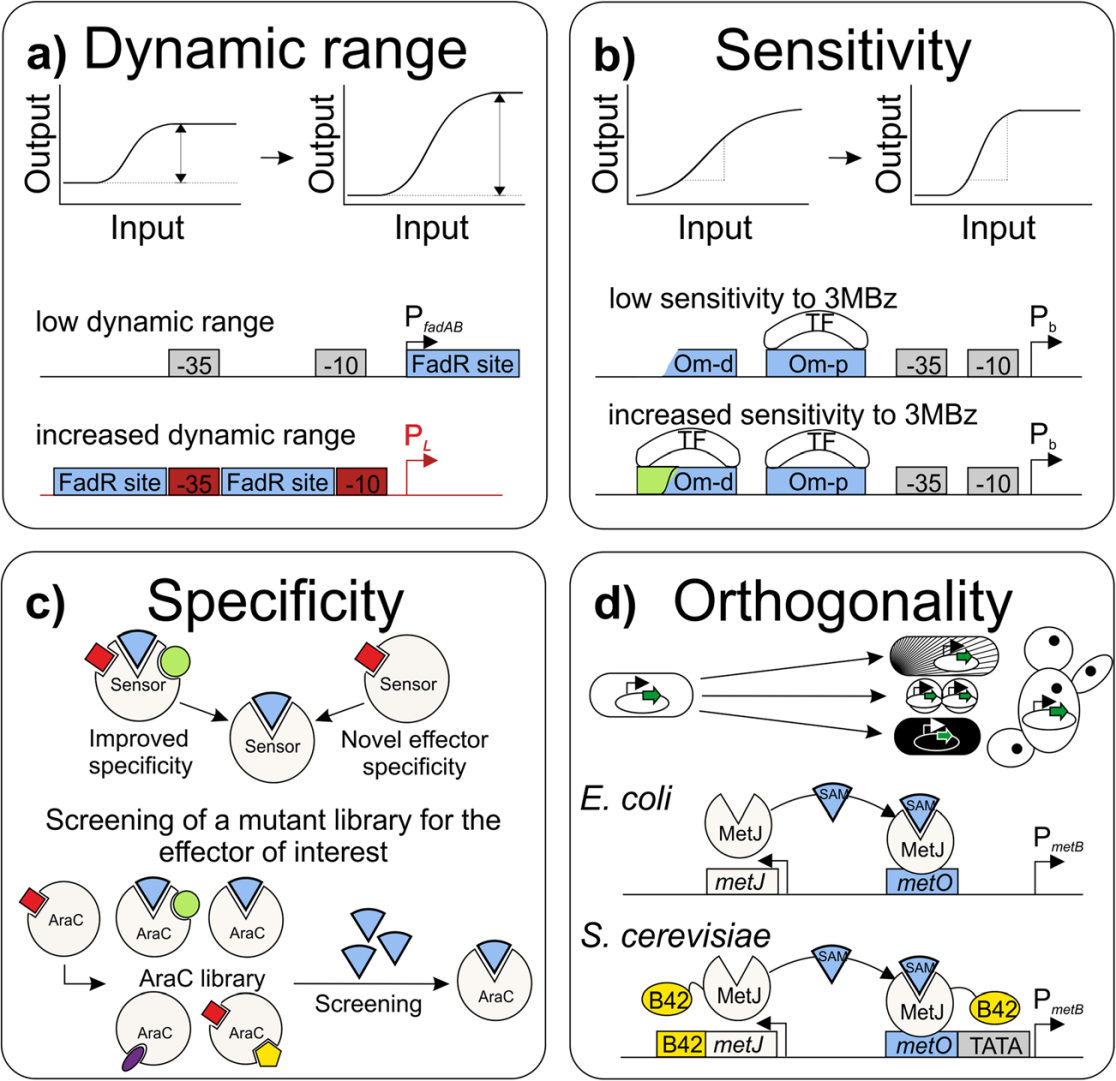
Examples of biosensor engineering for altered performance characteristics or orthogonal applications. **a** The dynamic range, describing the maximum fold change of a reporter output to a given input signal (Mustafi et al. 2015), was increased by introducing two FadR binding sites from the *fadAB* promoter into the strong lambda phage promoter P_L_ (Zhang et al. 2012). **b** To increase the sensitivity as rate of increase in reporter output (depicted by the slope of the transfer curve) to 3-methylbenzoate (*3MBz*), the truncated operator site *Omp-d* upstream of the operator site *Omp-p* in the P_*b*_ promoter was completed enabling the binding of two benzoate-binding transcription factors (*TF*) (Silva-Rocha and de Lorenzo 2012). **c** Furthermore, screening of an AraC mutein library for effectors of interest resulted in the identification of transcription factors with altered specificities (Tang and Cirino 2011; Tang et al. 2013). **d** The orthogonal transfer of biosensors to host organisms is challenging. Umeyama and co-workers equipped the S-adenosylmethionine (*SAM*)-responsive transcription factor MetJ of *E. coli* with the transcriptional activator domain B42 resulting in SAM detection in *S. cerevisiae* (Umeyama et al. 2013)

Although nature has evolved a variety of TF-promoter pairs, these sensor devices only exist for a limited number of cellular metabolites (Mustafi et al. 2015; Tang and Cirino 2011). As organisms tightly regulate their transcriptional machinery, endogenous promoter activity and its control are adapted to the organism’s purposes. For this reason, biosensors based on native transcription factors and promoters are often limited in sensitivity as well as the dynamic range, and are incompatible with non-native hosts (Blazeck and Alper 2013; Umeyama et al. 2013; Zhang et al. 2012, 2015). Furthermore, many biotechnological applications require the extension of promiscuous transcriptional regulators for specific or non-natural ligands (Looger et al. 2003; Schallmey et al. 2014). Due to the modular architecture of promoter regions (Blazeck and Alper 2013) and TFs (Galvao et al. 2007), engineering of biosensors for suitable performance characteristics becomes feasible (Fig. 3, Table 2). For example, Zhang and co-workers increased the dynamic range of a sensor system based on the fatty acid-sensing transcriptional regulator FadR about 1000-fold by the introduction of two copies of the FadR-DNA binding sequence into the strong phage lambda (P_L_) and phage T7 promoters (P_A1_) (Lutz and Bujard 1997; Zhang et al. 2012). By combining the FadR binding sites with a LacI operator site in the synthetic promoter, a tight regulation and induction by IPTG and fatty acids was accomplished, yielding a dynamic sensor-regulator system which enabled fatty acid ethyl ester production to be increased threefold (Fig. 3a) (Zhang et al. 2012).

**Table 2:** Examples for biosensor engineering

TF; source	Analyte	Host	Output	Characteristics/architecture
AraC-Idi_Synth_; *E. coli*	Isopentenyl diphosphate (lycopene)	*E. coli*	MutD5-mCherry	Sensor based on a synthetic TF composed of a isoprenoid binding domain and the DNA binding domain of AraC (Chou and Keasling 2013)
AraC-mev; *E. coli*	Mevalonate	*E. coli*	GFPuv	Screening of an AraC mutant library for a TF with a specific response towards mevalonate (mutated ligand binding site) (Tang and Cirino 2011)
AraC-Mut; *E. coli*	D-arabinose	*E. coli*	GFP	Screening of an AraC mutant library for a TF with a specific response towards D-arabinose (mutated ligand binding site) (Tang et al. 2008)
AraC-TAL; *E. coli*	Triacetic acid lactone	*E. coli*	GFP, LacZ	Screening of an AraC mutant library for a TF with a specific response towards triacetic acid lactone (mutated ligand binding site) (Tang et al. 2013)
BenR; *P. putida*	Benzoate, 3-methylbenzoate	*P. putida*	LuxCDABE	Introduction of a second operator motif into the promoter region increased specificity of the biosensor towards 3-methylbenzoate (Silva-Rocha and de Lorenzo 2012)
DcuS/EnvZ chimeric TCS; *E. coli*	Fumarate	*E. coli*	GFP	Chimeric TCS-based sensor for the extracellular sensing of fumarate (Ganesh et al. 2013)
GAL4-Idi_Synth_; *S. cerevisiae/E. coli*	Isopentenyl diphosphate (isoprenoids)	*E. coli*	Citrine	Sensor based on a synthetic TF composed of a isoprenoid binding domain and the DNA binding domain of GAL4 (Chou and Keasling 2013)
MalK/EnvZ chimeric TCS; *B. subtilis/E. coli*	Malate	*E. coli*	GFP	Sensor based on a chimeric TCS enabling the extracellular detection of malate by *E. coli* (Ganesh et al. 2015)
MetJ-B42; *E. coli*	S-adenosyl-methionine	*S. cerevisiae*	Venus, *HIS3*	Equipment of the *E. coli* TF MetJ with the transcriptional activation domain B42 results in the functional expression in *S. cerevisiae* (Umeyama et al. 2013)
PhlF; *E. coli*	2,4-Diacetylphloroglucinol	HEK293 cells	YFP	Equipment of the *E. coli* TF PhlF with eukaryotic-specific signals results in 2,4-diacetylphloroglucinol recognition in eukaryotic HEK293 cells (Stanton et al. 2014)
XylR; *P. putida*	3-Methyl-benzylalcohol m-xylene	*P. putida*	LuxCDABE	Equipment of the biosensor with a positive feedback loop and an attenuation mechanism shifted the specificity towards m-xylene (de Las Heras et al. 2012)

The modulation of the affinity and amount of TF binding sites can likewise contribute to the development of altered effector specificities and sensitivities (de Las Heras et al. 2012; Silva-Rocha and de Lorenzo 2012). For example, the TF BenR (AraC/XylS family) of *P. putida* KT2440 regulates P_b_ promoter activity by binding to the *Om-p* operator site in response to benzoate and with less efficiency to 3-methylbenzoate (3MBz) (Silva-Rocha and de Lorenzo 2012). Interestingly, the completion of a second truncated operator motif upstream of the *Om-p* site enhanced sensitivity of the sensor construct to 3MBz four- to fivefold (Fig. 3b) (Bintu et al. 2005a, b; Silva-Rocha and de Lorenzo 2012).

The modular architecture of regulators responding to effector molecules theoretically allows the development of any specificity and sensitivity (Fig. 3c) (Galvao and de Lorenzo 2006). Techniques generating genetic diversity, such as error-prone PCR (Wise and Kuske 2000), chemical and saturation mutagenesis (Tang and Cirino 2011; Tang et al. 2008, 2013) or computational modelling based on crystal structure data sets (Looger et al. 2003; Mandell and Kortemme 2009) contributed to the development of effector-molecule binding sites with altered or novel specificities (Galvao and de Lorenzo 2006). For example, the L-arabinose-response transcriptional regulator AraC was engineered by saturation mutagenesis to specifically respond to D-arabinose (Tang et al. 2008), to mevalonate (Tang and Cirino 2011) and to triacetic acid lactone (Tang et al. 2013). The de novo design of TF exhibiting the desired effector specificity was, furthermore, reported in a study by Chou and Keasling, who assembled the ligand binding domain of enzymes with the AraC DNA binding domains, yielding a synthetic transcription factor for the sensing of isopentenyl diphosphate (Chou and Keasling 2013). However, complex conformational changes occurring upon ligand binding and inter-domain interactions required for signal transduction make it more difficult to apply this strategy as a ubiquitous design approach.

The orthogonality of functional biological parts (e.g. promoters, coding sequences or terminators) still represents a major objective in the field of synthetic biology (Fig. 3d). Libraries of standardized modules (also designated as BioBricks) may contribute to facilitate the engineering of sensor devices. The functional transfer between organisms, however, still remains challenging. In an interesting study, Umeyama and co-workers fused the transcriptional regulator MetJ of *E. coli* to the transcriptional activation domain B42, yielding the synthetic TF MetJ-B42 which allows S-adenosylmethionine (SAM) sensing in the yeast *Saccharomyces cerevisiae* (Umeyama et al. 2013). Due to the extremely low diversity of regulatory proteins in mammalian cells, Stanton and co-workers supplied the PhlF repressor of *E. coli* with eukaryotic-specific signals (including a nuclear localization signal) and equipped regulated promoters with multiple operator sites resulting in 2,4-diacetylphloroglucinol recognition in HEK293 cells (Stanton et al. 2014). Although orthogonality still remains problematic, these examples show, however, that the transfer of sensor elements is feasible even across kingdom borders.

## Future prospects

TF-based biosensors have significantly contributed to a number of recent metabolic engineering approaches by improving production strains or by identifying non-producing subpopulations during bioprocesses (Fig. 2). However, a detailed molecular understanding of the observed phenotypic patterns during fermentation requires the establishment of highly sensitive *Omics* techniques interfacing with live cell imaging (e.g. in microfluidic chips) and cytometry analysis and cell sorting. Here, the combination of biosensors with next generation sequencing (e.g. RNA-seq) or high-resolution proteomics appears promising to reveal new insights into subpopulations and may support the identification of bottlenecks during bioprocesses.

Most biosensors reported to date are based on a small number of well-characterized TFs (Table 1). At this point, the screening of promoter libraries or transcriptome analysis using RNA-seq might contribute to harness still uncharacterized TFs for biosensor designs. However, accessibility to novel biosensor circuits and sensor components with altered effector specificities (e.g. to non-natural compounds) is key to a broad application in a wide variety of studies. As demonstrated by a number of studies, the modular design of TFs and their respective target promoters make a rapid design of novel circuits feasible (Fig. 3, Table 2). Despite this modularity and in-depth knowledge of the molecular basis, however, the design of synthetic regulatory circuits is not yet like a Lego set. To this end, future attempts must focus on the precise definition of highly orthogonal parts for sensor design and on the efficient generation of custom-made sensor domains with novel specificities and suitable characteristics (sensor transfer curves). Here, the combination of rational design and HT screening of mutant TF libraries appears most promising for efficient sensor design. Furthermore, the integration of synthetic biosensor circuits involves a metabolic burden for the host system which may affect productivity. Especially in the case of integral dynamic control circuits, the expression level of sensor components should be optimized to a minimum level, ensuring sensor functionality but minimizing interference with the host system.

TF-based biosensors have the potential to revolutionize recent strategies in biotechnological strain development. However, several studies still remain at the level of sensor construction and proof-of-principle applications. To enhance the availability of sensors with appropriate characteristics, more studies are required to establish efficient workflows for biosensor design. Altogether, these efforts should aim to enable an application-oriented construction of biosensors to allow the rapid engineering of required circuits meeting the needs of the particular metabolic engineering purpose.
